# Human chitinases and chitinase-like proteins as emerging drug targets – a medicinal chemistry perspective

**DOI:** 10.1039/d4md01050g

**Published:** 2025-04-24

**Authors:** Önder Kurç, Nick Rähse, Holger Gohlke, Jonathan Cramer

**Affiliations:** a Heinrich Heine University Düsseldorf, Faculty of Mathematics and Natural Sciences, Institute for Pharmaceutical and Medicinal Chemistry Universitätsstr. 1 40225 Düsseldorf Germany gohlke@uni-duesseldorf.de jonathan.cramer@hhu.de; b Forschungszentrum Jülich, Institute of Bio- and Geosciences (IBG-4: Bioinformatics) Wilhelm-Johnen-Str. 52425 Jülich Germany

## Abstract

Human chitinases and chitinase-like proteins (CLPs) provide the immune system with the ability to recognize or process chitin originating from chitinous pathogens. In addition to their role in host defense, most members of this protein family have evolved pleiotropic cellular effector functions broadly related to immune homeostasis, cell proliferation, and tissue remodeling. This wide-ranging ability to modulate crucial cellular processes proceeds *via* the activation of cellular signal transduction cascades and appears to be fully independent of chitin recognition. Dysregulation of chitinase/CLP functions has been linked to a plethora of inflammatory diseases, such as allergic airway diseases and asthma, fibrosis, as well as cancer. This fact predetermines certain members of this protein family as prime targets for pharmacological intervention. Here, we provide an extensive review of medicinal chemistry efforts targeting the most widely studied members of the human chitinase/CLP family, namely acidic mammalian chitinase (AMCase), chitotriosidase (CHIT1), and chitinase-3-like protein 1 (CHI3L1/YKL-40).

## Abstract 2

Human chitinases and chitinase-like proteins are attractive drug targets. This review focuses on medicinal chemistry efforts directed at acidic mammalian chitinase (AMCase), chitotriosidase (CHIT1), and chitinase-3-like protein 1 (CHI3L1/YKL-40).

## Graphical abstract

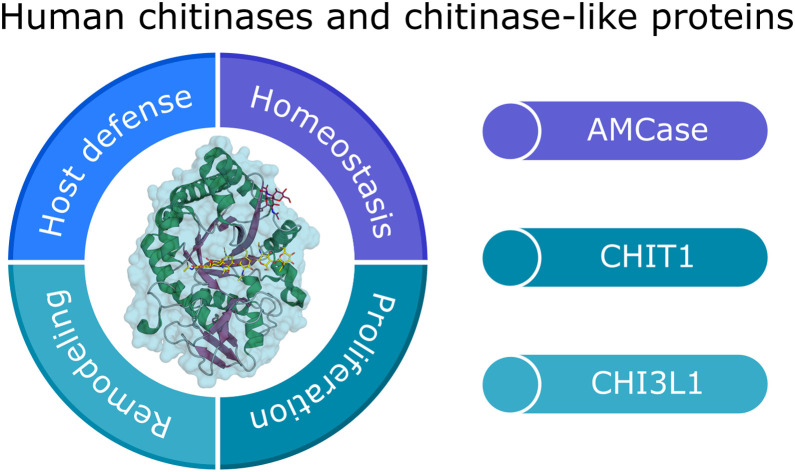

## Human chitinases and chitinase-like proteins

Chitin is a natural linear polysaccharide consisting of repeating units of β-1,4-linked *N*-acetyl-d-glucosamine (GlcNAc). Chitin and its derivatives are the main structural components of cell walls in fungi and the shells of crustaceans and insects, which renders chitin the second most abundant polysaccharide in nature. Chitin-containing organisms employ chitinases for endogenous chitin remodeling during developmental processes, while many microorganisms also utilize these enzymes to degrade exogenous chitin as a nutritional substrate, making them a promising target for combating pathogenic fungi and controlling agricultural pests.^1,2^ It is, however, not endogenous to plants and vertebrates. In this context, many organisms can sense the polymer as a marker for infection by chitin-containing pathogens such as parasitic-fungi and arthropods. The human innate immune system can recognize chitin through pattern recognition receptors, such as TLR2 or dectin-1.^3^ In addition, secreted chitinases are produced in tissues prone to encountering environmental pathogens.

**Fig. 1:**
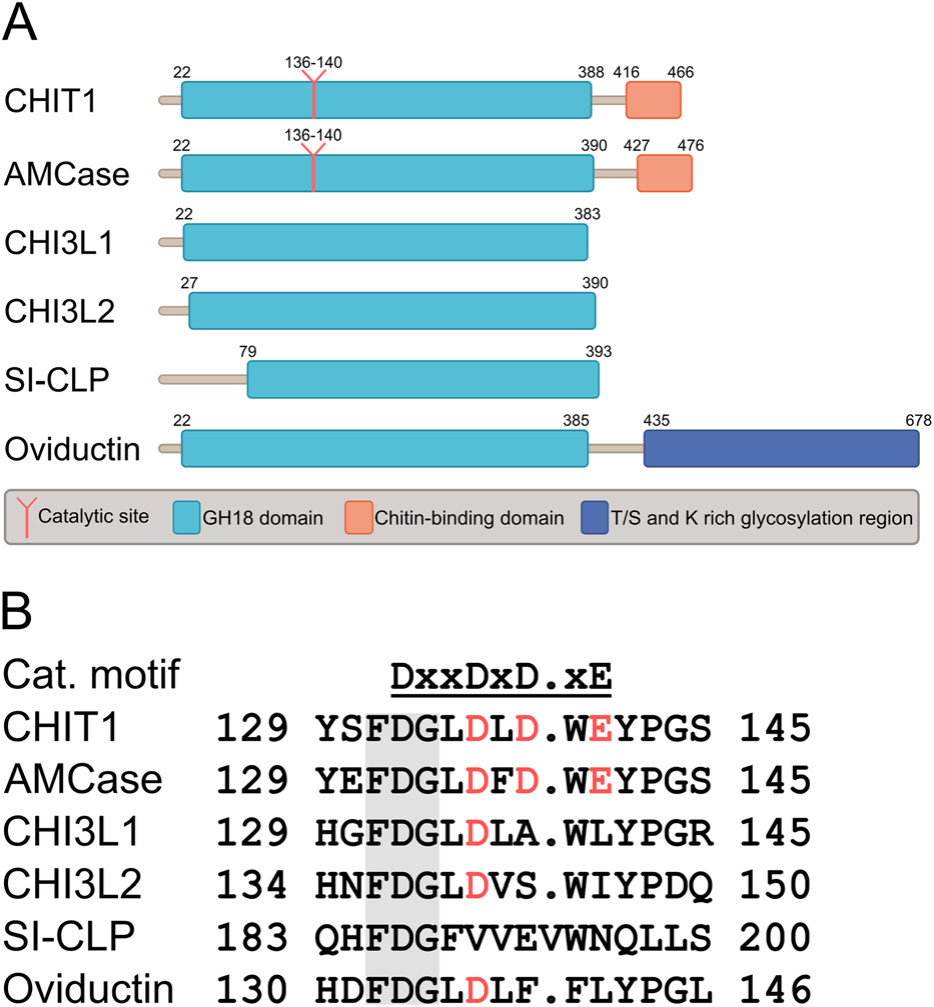
A) Domain organization and B) sequence alignment of the catalytic sites of human chitinases and CLPs.

Two enzymatically active chitinases from the glycosyl hydrolase 18 (GH18) family, chitotriosidase (CHIT1) and acidic mammalian chitinase (AMCase, CHIA), have been described and characterized in humans.^4^ These enzymes feature a secondary chitin binding domain in addition to the canonical GH18 domain (Fig. 1A).^1^ Their catalytic activity is conveyed by a conserved DXXDXDXE motif (Fig. 1B). In the context of human diseases, CHIT1 and AMCase have been linked to lysosomal storage disorders,^5^ sarcoidosis,^6,7^ and respiratory system diseases including asthma,^8,9^ chronic obstructive pulmonary disease,^10,11^ and idiopathic pulmonary fibrosis.^12,13^

**Fig. 2:**
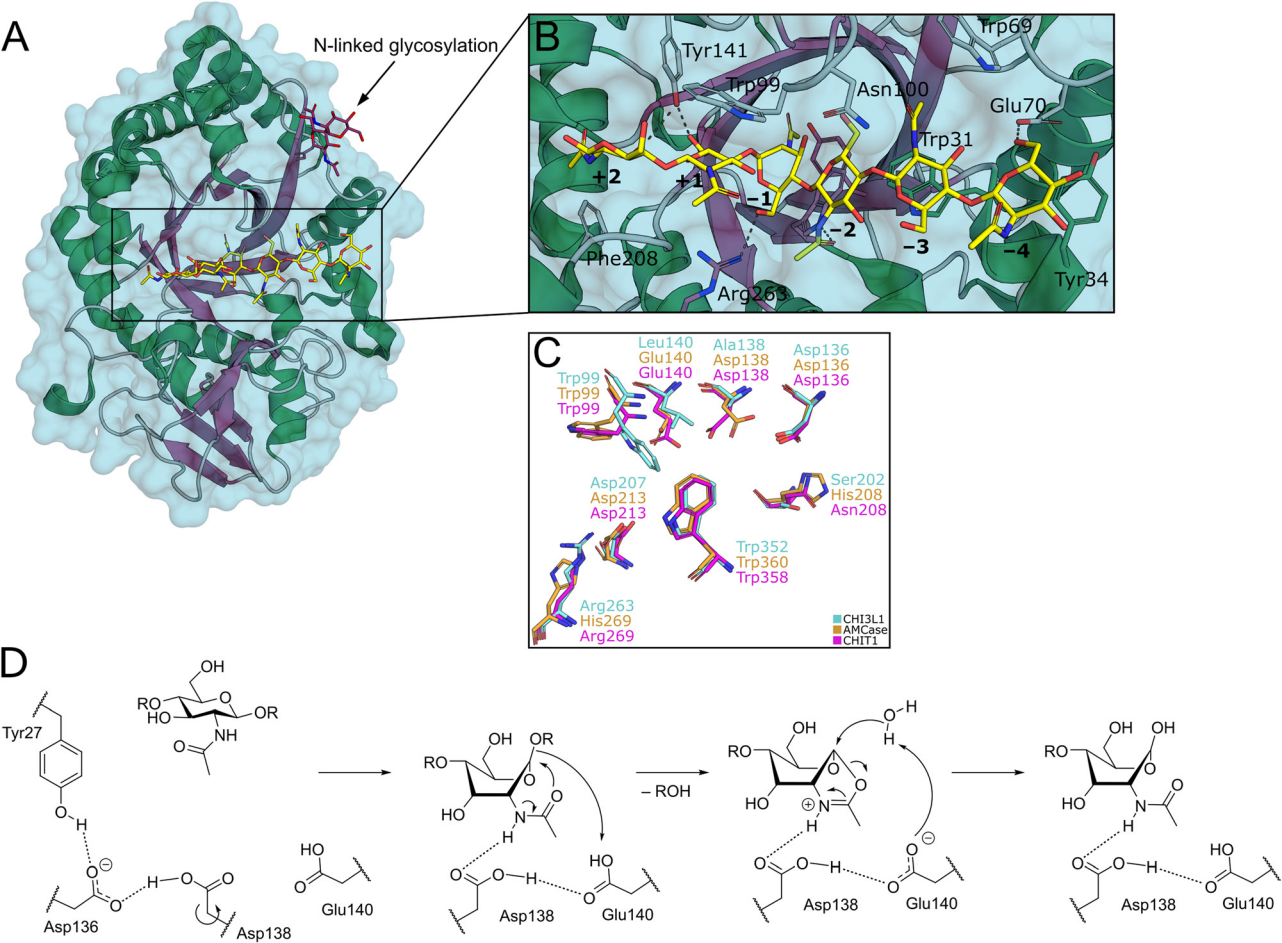
A) Crystal structure of CHI3L1 as an example for GH18 proteins (PDB ID 1HJW). B) Close-up view of the chitin binding cleft with subsites +2 to −4 harbouring a COS ligand. C) Superposition of the −1 to +1 subsites of CHI3L1, AMCase, and CHIT1 (PDB ID 1NWR, 4WKA, and 3FXY). D) Substrate-assisted mechanism of chitin hydrolysis by GH18 chitinases (CHIT1 numbering).

Structurally, the catalytic domain of GH18 family enzymes is characterized by a (β/α)_8_ triosephosphate isomerase (TIM)-barrel fold (Fig. 2A).^1^ An extensive linear cleft spanning across the central β-barrel can bind chitin oligosaccharides (COS) of variable length. The GlcNAc monomers are accommodated in subsites, which are systematically numbered according to their relation to the cleavage site (Fig. 2B).

By this convention, chitin hydrolysis occurs between subsites −1 (non-reducing end) and +1 (reducing end). COS are typically bound through hydrogen bonds with their polar hydroxyl groups and acetamide moieties, as well as CH–π interactions between the apolar carbohydrate β-face and tryptophan residues, which are abundant in the chitin binding site. The catalytic mechanism of GH18 enzymes proceeds under retention of stereochemistry by a substrate-assisted double inversion mechanism.^1^ The ^4^C_1_-chair equilibrium conformation in solution is distorted into an energetically unfavorable ^1,4^B-boat conformation upon binding to the active site of the enzyme. After protonation of the glycosidic oxygen atom, this change in ring puckering enables an intramolecular attack of the acetamide carbonyl oxygen on the anomeric center, resulting in the formation of a charged oxazolium intermediate, which is subsequently attacked by a water molecule to release the product as a hemiacetal (Fig. 2D).^14,15^

In addition to the enzymatically active chitinases, several chitinase-like proteins (CLPs) have been discovered in humans, namely chitinase-3-like protein 1 (CHI3L1, YKL-40, HCgp-39), chitinase-3-like protein 2 (CHI3L2, YKL-39), stabilin-1 interacting CLP (SI-CLP, CHID1), and oviductin.^16^ While CLPs are largely homologous with active chitinases on a sequence and structural level (Fig. 1A), they lack enzymatic function. This loss of enzymatic function has been linked to mutations in the DXXDXDXE motif conveying catalytic activity (Fig. 1B).^15^ Yet, CLPs still display affinity towards chitin, which is mediated by their intact chitin binding site. Their biological function, however, is not related to chitin processing. Instead, CLPs exert context-dependent effector functions linked to immune homeostasis, cell proliferation and migration, tissue remodeling, and other processes.^17^ These pleiotropic functions are mediated by interactions with various cellular receptors and subsequent activation of intracellular signaling cascades. In a disease context, CLPs have been implicated in oncogenesis, respiratory diseases, and other disorders.^17^ As a consequence, human chitinases and CLPs have emerged as promising targets for pharmacotherapy. In this review, we focus on recent progress in the development of drugs targeting the most well-studied members of the human chitinase/CLP family, namely AMCase, CHIT1, and CHI3L1.

## Natural product chitinase inhibitors

**Fig. 3:**
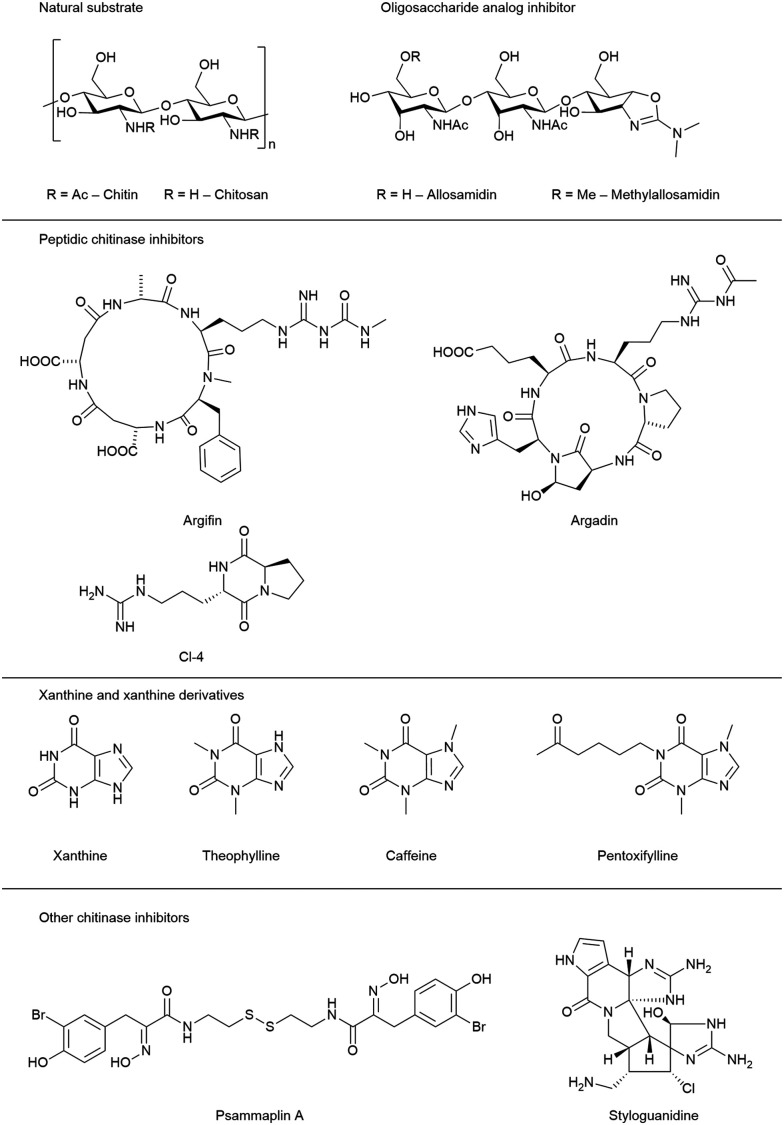
Chemical structure of natural product chitinase inhibitors.

Several natural products of high structural diversity have been identified as broad-spectrum chitinase inhibitors (Fig. 3). This includes the oligosaccharide analog allosamidin,^18^ cyclic peptides (argifin,^19,20^ argadin,^21^ Cl-4 (cyclo-l-Arg-d-Pro)),^22^ as well as xanthine and xanthine derivatives (caffeine, theobromine, theophylline, and the synthetic analog pentoxyfylline).^23^ Additionally, psammaplin A^24^ and styloguanidine^25^ were demonstrated to exhibit potent inhibitory activity against chitinases. These compounds are produced by a wide range of organisms, including soil and marine microbes, fungi, plants, and animals.^20^

Crystal structures of various GH18 chitinases in complex with allosamidin and its derivatives have revealed that the allosamizoline moiety occupies the −1 subsite, representing a non-hydrolysable mimetic of the oxazolium intermediate (Fig. 2D). Argifin, argadin, and Cl-4 similarly target the active site: argifin engages the −1 subsite through its *N*-methyl carbamoyl-derivatized arginine side chain, whereas argadin and Cl-4 utilize the histidine side chain or diketopiperazine heterocycle for binding at this position, respectively.^26,27^ Additionally, the basic imidazolopyrimidine heterocycle of xanthine derivatives also binds to the catalytic site in the −1 pocket.^23^

Due to a combination of structural complexity, lack of selectivity, unfavorable physicochemical properties, or low potency, none of these compounds were seriously considered for further (pre-)clinical development. Nevertheless, natural chitinase inhibitors can offer valuable insight into the catalytic mechanism and represent potential leads for drug discovery and pest control.^28–31^

## Acidic mammalian chitinase

Acidic mammalian chitinase (AMCase, CHIA) is commonly expressed by epithelial cells, neutrophils, and macrophages in the brain, eyes, stomach, lung, kidneys, and nose.^32–34^ Its name originated from the ability of the enzyme to resist acidic environments. Consequently, the enzyme displays remarkable stability under acidic conditions, a property that is considered to be conveyed by a central histidine residue in position 187,^35^ and resistance against proteolytic cleavage by gastric proteases.^36^ In addition to its role in the protection against chitinous pathogens, its paralogs mediate the digestion of chitin in insectivore mammals like bats and mice. This finding has been previously linked to the ability of early primates to utilize chitin as a source of nutrition, which was lost during the evolutionary development of humans.^37^

In nasal glands, AMCase is exponentially upregulated when the host is confronted with chitin-containing pathogens.^38^ Similarly, AMCase expression in the lungs is induced upon contact with chitin-containing allergens, such as house dust.^39^ This allergen-induced overexpression of AMCase might contribute to increased allergic airway inflammation.^40^ The chitinolytic activity of AMCase is regulated by environmental stimuli, such as ionic strength and pH.^40^ With a pH optimum between 4 and 5, AMCase is hypothesized to be predominantly active in the pathologically acidified airways of asthma patients, as well as the acidic stomach environment.^8,9^

Beyond its role in host defense, AMCase is associated with various other functions related to cell proliferation and survival, as well as tissue remodeling.^40^ Cytoprotection of airway epithelial cells has been linked to an activation of the PI3K/Akt pathway by AMCase.^41^ This function was not related to the chitinolytic activity of the enzyme, as a recombinant catalytically inactive AMCase mutant retained the ability to protect epithelial cells from growth factor withdrawal and Fas ligand-induced apoptosis. This indicates that AMCase might enact its non-chitin-related effector functions by engaging yet unknown cellular receptors in analogy to CLPs (see section on CHI3L1 below). Notably, the cytoprotective effect of AMCase was abrogated by treatment with the pan-chitinase inhibitor allosamidin, even when a catalytically inactive mutant protein was employed. This key insight demonstrates that even non-chitin-related functions of chitinases can be modulated by molecules binding to the chitin binding site, suggesting a potential allosteric coupling between this region and distal secondary binding sites for its cellular receptors. Whereas a blockade of AMCase by allosamidin or an anti-AMCase antibody ameliorated airway inflammation and hyper-responsiveness in a non-chitin-dependent allergy model,^8^ the opposite observation was made in a chitin-dependent model of pulmonary inflammation.^42^ This points to the detrimental role of a misguided and overshooting immune response in the absence of chitinous pathogens. Despite the strong evidence of AMCase involvement in the pathology of allergic airway diseases, a contrasting report suggests a less prominent role of the enzyme.^43^

### Discovery of small-molecule AMCase inhibitors

**Fig. 4:**
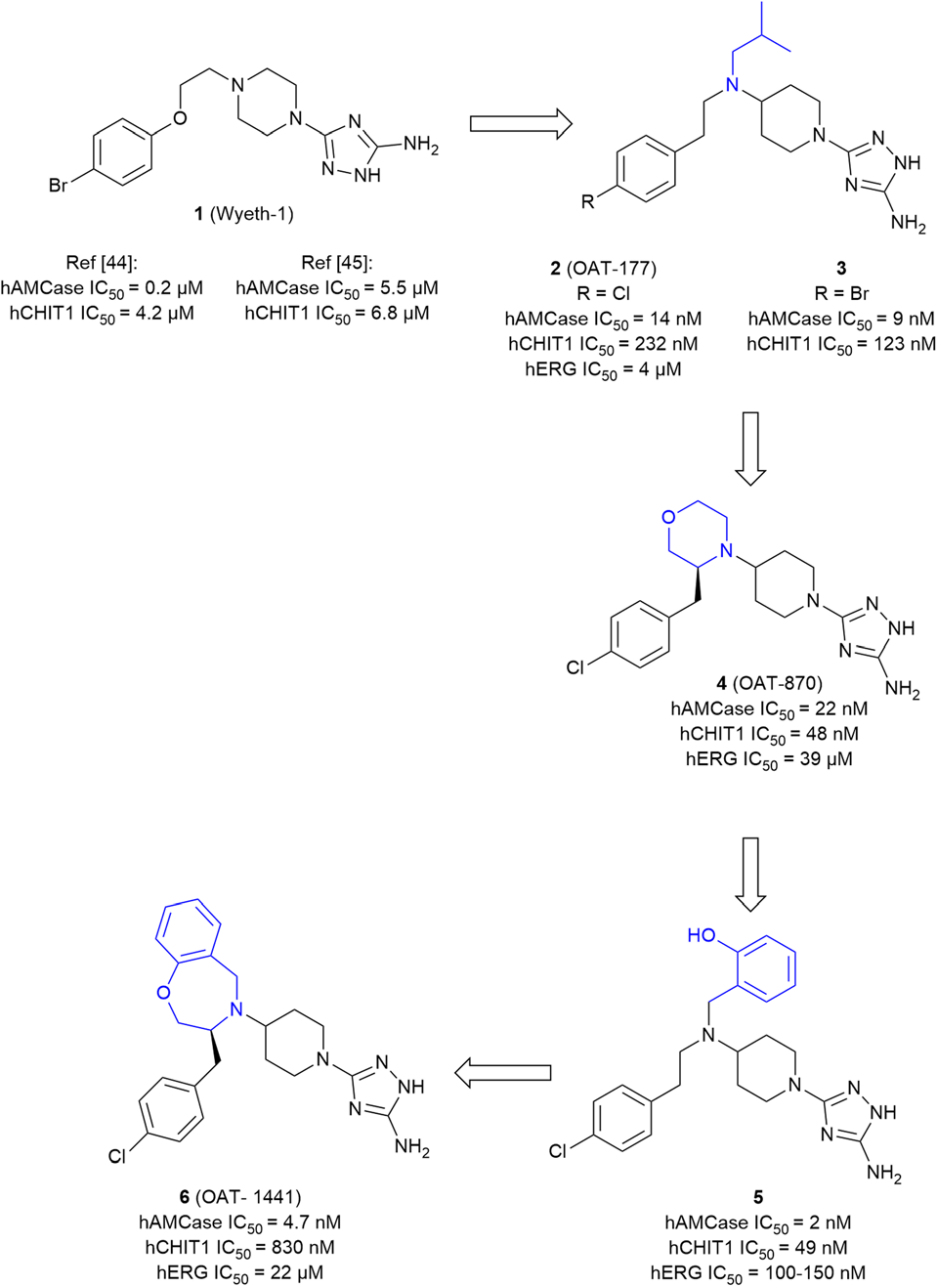
Evolution of aminotriazole-based AMCase inhibitors.

**Fig. 5:**
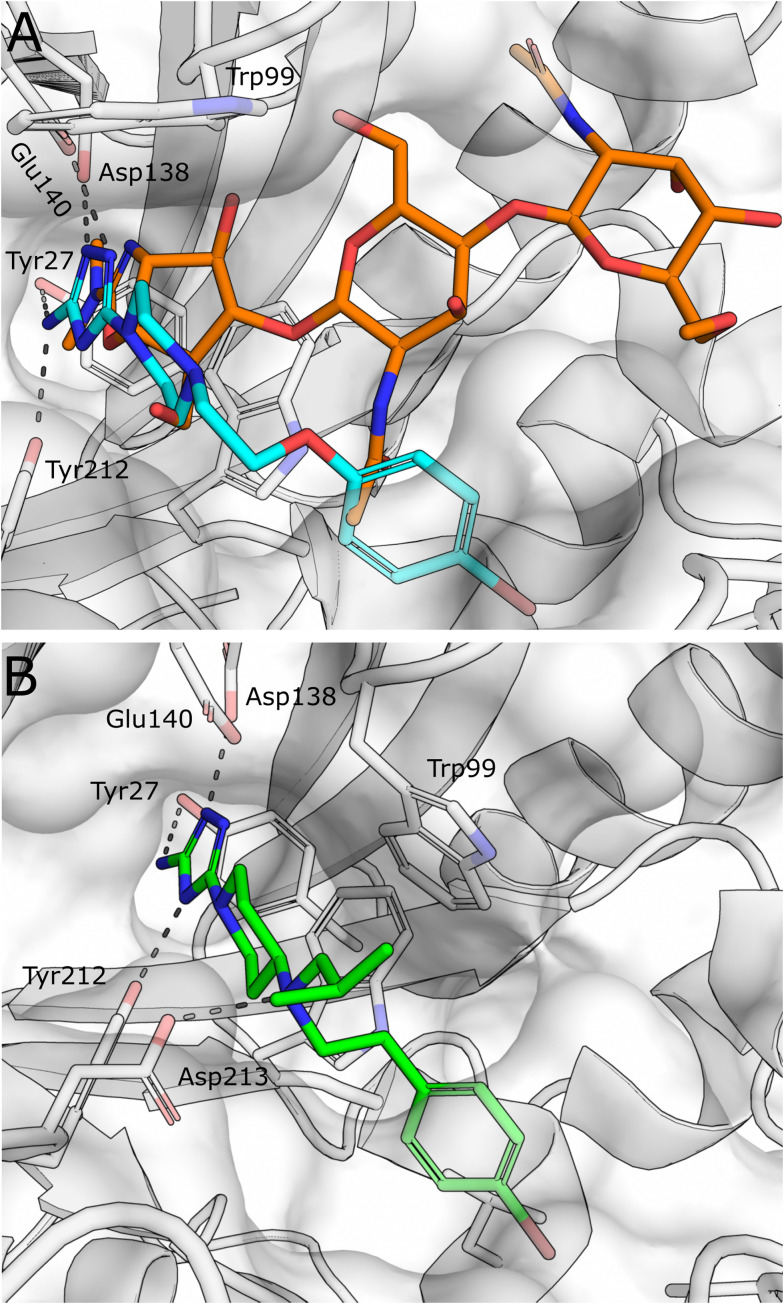
A) Superposition of X-ray crystal structures of AMCase in complex with methylallosamidin (PDB ID 3FY1, orange) and 1 (PDB ID 3RM4, cyan). B) X-ray crystal structures of AMCase in complex with 3 (PDB ID 5NRA).

Given the therapeutic potential of the enzyme, the development of small molecule AMCase inhibitors was initiated by an industry-led discovery campaign.^44^ Initially, a library consisting of 466 000 compounds was screened *via* a high throughput enzyme assay (HTS). The resulting list of potential binders was then amended with hits from an orthogonal virtual screening (VS) employing a library of 150 000 additional molecules. In a complementary fragment-based design approach, a library of 1045 fragments was screened by STD-NMR. With hit rates ranging from 0.18% (HTS) to 0.96% (FBD) and 1.96% (VS) after validation, the complimentary screening campaigns identified the high-affinity inhibitor 1 (Wyeth-1, Fig. 4) with an IC_50_ value of 210 nM (enzyme assay) and a *K*_D_ of 1.69 μM (SPR). In the crystal structure of the complex, 1 was found to closely mimic the binding mode of allosamidin (Fig. 5A). The aminotriazole moiety of 1 was accommodated in the active site of the enzyme, interacting with the catalytic residues Asp138 and Glu140, as well as Tyr212 and Tyr27. In addition, the bromobenzene moiety of 1 engages in apolar interactions in a nearby hydrophobic region of the chitin binding cleft. To elucidate the effectiveness of AMCase inhibition in allergic airway disease, mice were challenged with house dust mite and cockroach antigens. The chitinolytic activity measured in bronchoalveolar lavage fluid (BALF) was found to be significantly reduced (43% reduction) after treatment with 1, compared to a reduction of 60% after treatment with dexamethasone.

Building on these results, Mazur *et al.* embarked on a structure-guided optimization campaign focusing on modifications of the central piperazine moiety.^45^ The heterocycle was converted to an aminopiperidine decorated with bulky and hydrophobic aryl and alkyl groups. This effort led to the discovery of 2 (OAT-177). Crystallography revealed that the modifications successfully enhanced peripheral hydrophobic contacts of the related analog 3 in the chitin binding cleft, while retaining the strong polar interactions of the aminotriazole moiety in the active site (Fig. 5B). An additional salt bridge with Asp213 was enabled by shifting the basic nitrogen to the exocyclic position. This resulted in an increased potency (IC_50_ = 14 nM) compared with the parent molecule 1, which was reported with a weaker affinity of IC_50_ = 5.5 μM in this study. Notably, an approximately 16-fold selectivity *versus* the closely related chitinase CHIT1 was achieved (see Table 1 for a summary of all discussed AMCase/CHIT1 inhibitors). This observation was linked to a difference in the interaction of the aminotriazole moiety, which forms a direct contact with Glu140 in the crystal structure with AMCase, whereas a weaker water-mediated interaction is observed in CHIT1.

**Table 1:** Summary of biological assay data for hAMCase and CHIT1 inhibitors

Compound	hAMCase^a^ [nM]	hCHIT1^a^ [nM]	hERG^a^ [μM]	Ref.
1 (Wyeth-1)	200	4200	N/A	44, 45
2 (OAT-177)	14	232	4	45
3	9	123	N/A	45
4 (OAT-870)	22	48	39	47
5	2	49	0.1–0.15	48
6 (OAT-1441)	4.7	830	22	48
7 (bisdionin B)	90 000	110 000	N/A	49
8 (bisdionin C)	3400	8300	N/A	49
9 (bisdionin F)	900	17 000	N/A	50
10	13 000	10 000	N/A	28
11	18 000	>50 000	N/A	28
12	2000	>12 000	N/A	28
13	1590 (mAMCase)	7600 (mAMCase)	N/A	79
14 (OAT-2068)	84	1250	2.4	79
15 (OATD-01)	9	23	23	80
16	6800 (*K*_i_)			82
17	396 000 (*K*_i_)	49 (*K*_i_)		82

a The reported data are IC_50_ values, unless otherwise noted.

An investigation of the *in vivo* pharmacokinetic (PK) properties of 2 revealed an overall favorable profile with an acceptable oral bioavailability (52%) dominated by hepatic first pass and low renal clearance. However, potential off-target toxicity of 2 through a considerable blockade of the hERG potassium channel (IC_50_ = 4 μM) and strong inhibition of serotonin and dopamine transporters (95% inhibition at 10 μM) remained a serious concern. In a mouse model of allergic airway disease, oral treatment with 2 led to a significant reduction of chitinolytic activity in BALF, concomitant with a reduction in total leukocyte and eosinophil numbers in particular. In addition, IgE concentration in plasma was markedly reduced in the treated animals.

In a later study, 2 was investigated in the context of gastrointestinal inflammatory diseases employing a dextran sulfate sodium-induced mouse model of colitis.^46^ The effect of the compound was evaluated by the assignment of a macroscopic score, measurement of colon length, colon weight, and myeloperoxidase levels, which were known to be affected by an inflammatory response. As a result, a significant anti-inflammatory effect and decrease in expression of AMCase was observed with a low dose of 30 mg kg^−1^. When the dose was increased to 50 mg kg^−1^ and higher, the protective effect of 2 was reversed and an exponentially increased expression of CHIT1 was observed. Subsequent lead optimization was driven by the ambition to convert the moderately selective AMCase inhibitor 2 into a dual AMCase/CHIT1 inhibitor while simultaneously reducing hERG inhibition.^47^ For this, the hydrophobicity of the compound was diminished by an exchange of the apolar isobutyl substituent against more polar moieties. Simultaneously, the outright integration of this region into a saturated heterocycle was attempted to restrict the conformational flexibility of the molecule. Whereas the former strategy was met with limited success, the introduction of a substituted morpholine in this position (→4, OAT-870) successfully retained high activity against both chitinases, while simultaneously reducing hERG inhibition by a factor of 10 (IC_50_ = 39 μM, FP assay), although a higher activity was later observed in a patch-clamp assay (IC_50_ = 12 μM). Despite a successful optimization of some properties, near-complete dopamine transporter inhibition remained a significant challenge. 4 displayed good oral bioavailability and low clearance in mice and was advanced into animal studies, where it revealed a sizeable reduction of CD45^+^ leukocytes in BALF of allergen-challenged mice.

Reverting to the concept of AMCase selective inhibitors featuring purely hydrophobic substituents on the central aminopiperidine, a series of decorated benzyl moieties was introduced in this position.^48^ This effort yielded highly potent AMCase inhibitor 5 with single digit nM activity and improved selectivity against CHIT1. However, this gain in potency went along with a strongly unfavorable pharmacokinetic (PK) profile characterized by potent hERG inhibition (IC_50_ = 100–150 nM) and poor microsomal stability (*t*_½_ < 1 h). Inspired by the previous success originating from a reduction of the conformational flexibility, the lead structure was converted into a bicyclic benzoxazepine (6, OAT-1441). This modification resulted in a 10-fold increase of AMCase/CHIT1 selectivity, while retaining low nM potency. Off-target toxicity was simultaneously reduced to a more acceptable level (hERG: IC_50_ = 22 μM (FP assay), IC_50_ = 23 μM, (patch-clamp assay); dopamine transporter: 37% inhibition at 10 μM). 6 displayed a generally favorable pharmacokinetic profile characterized by an oral bioavailability of 41% and moderate plasma clearance.

**Fig. 6:**
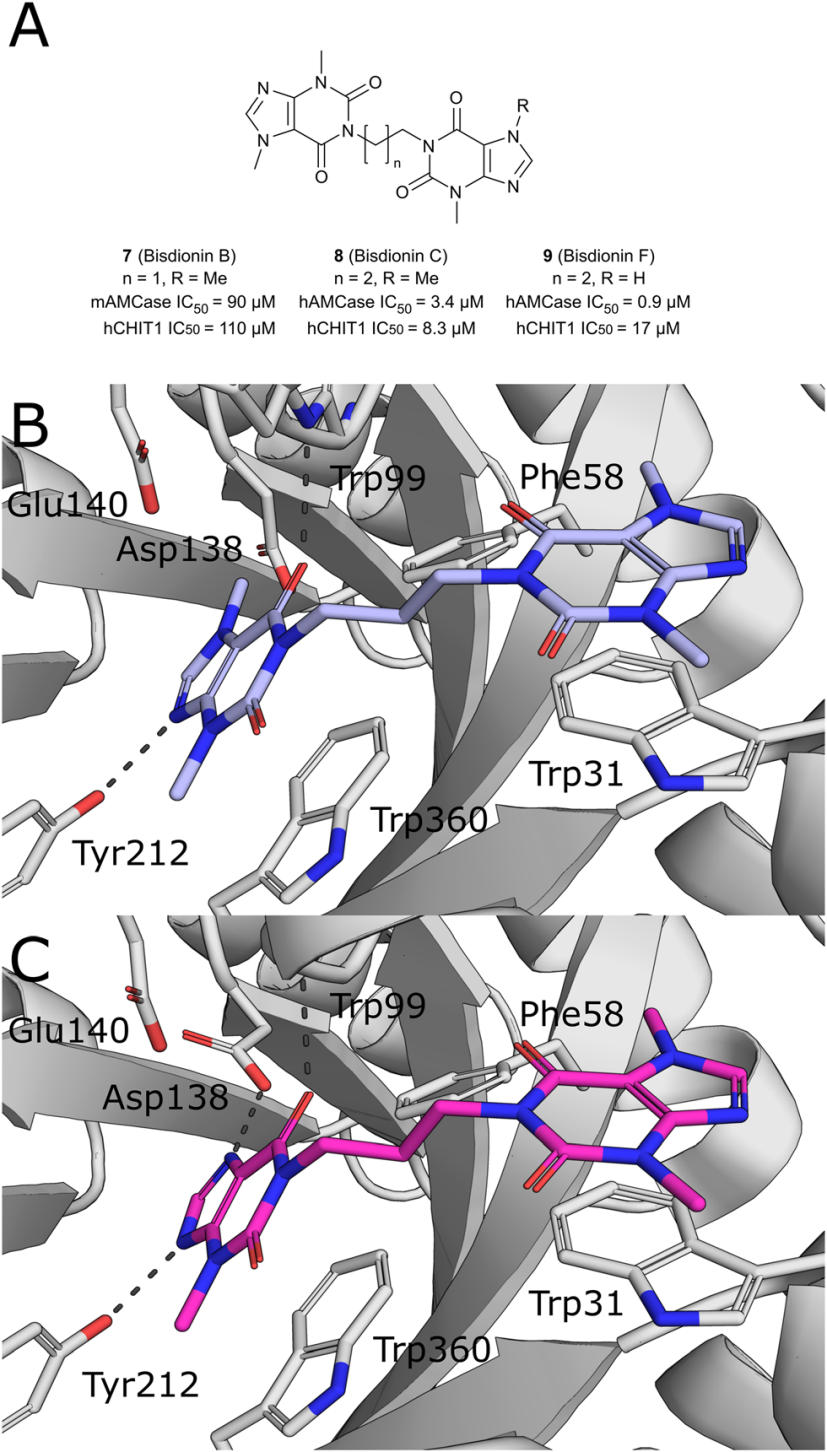
A) Chemical structures of bisdionins C, D, and F. B) X-ray crystal structures of AMCase in complex with bisdionin C (8, PDB ID 2YBT) and C) bisdionin F (9, PDB ID 2YBU).

Starting from the crystallographic observation that the chitin binding site of GH18 chitinases can accommodate two xanthine derivative molecules simultaneously, Schüttelkopf *et al.* disclosed the discovery of dimeric xanthine derivatives bisdionin B/C (7–8) as moderately potent AMCase inhibitors with no selectivity against CHIT1 (Fig. 6A).^49^ A detailed analysis of the bisdionin C/AMCase crystal structure revealed that *N*7-methylation in bisdionin C imposed an unfavorable conformational change of the Asp138 side chain, which was not observed in the structure with CHIT1 (Fig. 6B).^50^ Demethylation of this position yielded bisdionin F (9), which favorably engaged Asp138 (Fig. 6C), showed greatly improved AMCase inhibition (IC_50_ = 0.9 μM) and approximately 20-fold selectivity against CHIT1. In a mouse model of allergic airway inflammation, the lung homogenate of OVA-challenged mice showed reduced chitinase activity after treatment with 9. Analysis of the cellular infiltrate into BALF revealed a significant depletion of lymphocytes and eosinophils and, unexpectedly, an increase in the number of neutrophils in the treated animals. In addition, chitinase inhibition resulted in an altered expression of genes associated with tissue remodeling, such as matrix-metalloprotease 12, Ym1, and tissue inhibitor of metalloproteinases 1.

**Fig. 7:**
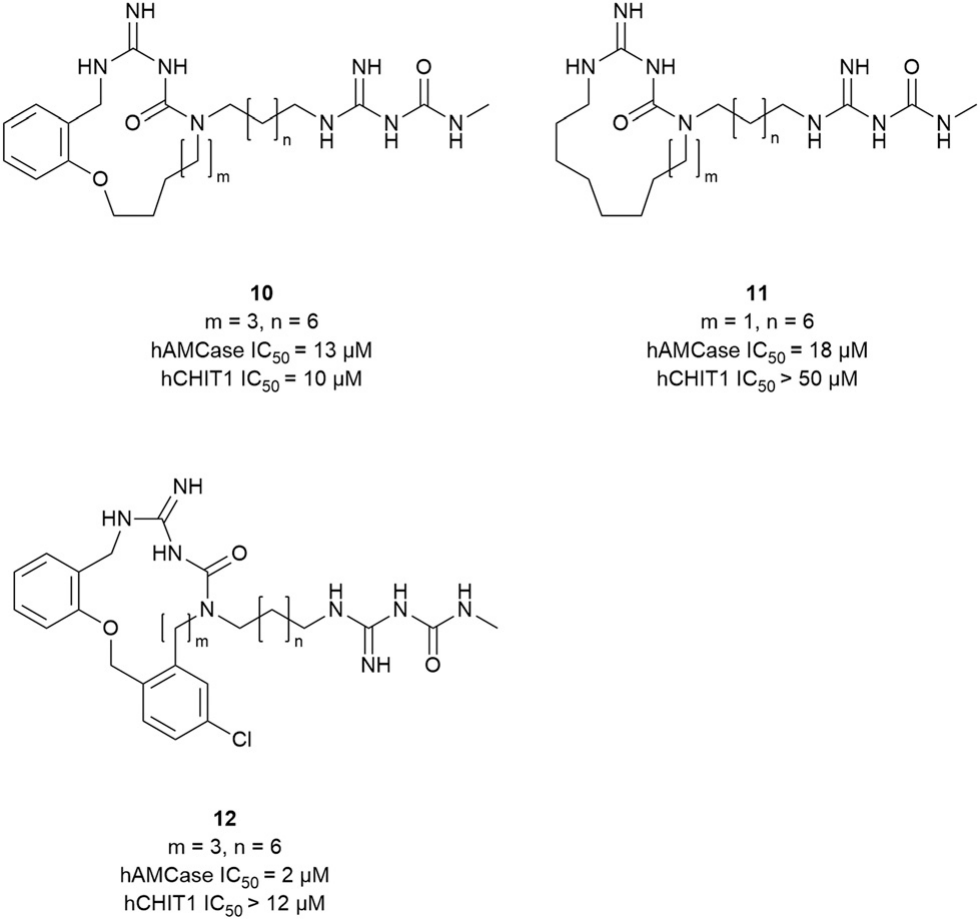
Macrocyclic chitinase inhibitors.

Taking inspiration from the peptidic chitinase inhibitor argifin, Balestri *et al.* developed novel macrocyclic amidinoureas as inhibitors of AMCase and CHIT1.^28^ In an attempt to explore macrocycle flexibility and potential aromatic interactions, several series differing in ring size and nature of the ring-forming linkage were synthesized (10–12, Fig. 7). This effort yielded the dibenzena-cyclophane 12 as the most potent compound of the series, which inhibited AMCase with a *K*_i_ of 2 μM and a selectivity index of 6*versus* CHIT1. Relying on docking studies, the authors concluded that the exocyclic amidinourea moiety interacted with the active site residues, whereas the macrocyclic portion of the molecule engaged in additional interactions within the chitin binding cleft. Preliminary ADME studies with the most potent compounds revealed good microsomal stability and led to their classification as BCS class III compounds (high solubility, low permeability).

## Chitotriosidase

Human chitotriosidase (hCHIT1) was the first mammalian chitinase to be identified.^51,52^ In addition to its endochitinase activity,^53^ it displays trans-glycosylation activity at excess substrate concentration.^54^ As a component of the innate immune system,^55^ CHIT1 plays a protective and regulatory role against the susceptibility to infection by chitin-containing pathogens.^56–58^ Over the past few decades, numerous studies have emerged detailing the differential regulation of CHIT1 expression during specific immunological activities, with much of this research focusing on its association with diseases characterized by inflammation and remodeling.^59^ Interestingly, different Caucasian populations show a high frequency of a CHIT1 gene mutation, characterized by a 24-bp duplication in exon 10, which results in an abnormally spliced mRNA encoding an enzymatically inactive protein lacking 29 amino acids.^60^ Conversely, the low prevalence of this polymorphism in African populations living in malaria-endemic areas suggests that the wild-type CHIT1 gene has been maintained for its role in providing innate protection against parasitic diseases like malaria.^61^ These findings suggest that CHIT1 may have become functionally redundant in regions with lower endemic parasitic burdens or even detrimental in the context of certain inflammatory and degenerative disorders.

CHIT1 is widely distributed in human tissues, such as salivary gland, liver, thymus, and spleen, with the lung and stomach showing the highest mRNA levels.^62^ It is produced at minimal levels in healthy persons but is highly expressed in response to several pro-inflammatory signals in activated macrophages,^5,26^ and in other cells such as neutrophils,^63^ Kupffer cells,^64^ and bronchial epithelial cells.^11^ The elevated secretion of CHIT1 in serum is implicated as a diagnostic biomarker for Gaucher's disease (GD).^5^ Patients with GD can exhibit a 10- to 1000-fold increase in CHIT1 activity in the blood, which appears to correlate with disease severity.^5,65^ This makes CHIT1 a potential target for therapeutic strategies, as well as an indicator for monitoring treatment effects. Beyond GD, an increase in CHIT1 enzymatic activity has been observed in other lysosomal storage disorders and conditions such as Niemann–Pick disease, atherosclerosis, and malaria.^66–68^ In addition, numerous biomarker studies from patients with chronic respiratory diseases, including sarcoidosis, idiopathic pulmonary fibrosis (IPF), chronic obstructive pulmonary disease (COPD), and asthma, have reported high expression or activity of the enzyme in diagnostic fluids.^11,69–71^ Moreover, CHIT1 upregulation has been linked to several neurological diseases, including Alzheimer's disease,^72^ amyotrophic lateral sclerosis (ALS),^73^ and multiple sclerosis (MS).^74^ While CHIT1 is well-established as a clinical marker, its precise role in regulating inflammation, immune responses, and its direct contribution to the pathogenesis of all of these diseases, remains to be fully elucidated.

Few mechanistic studies have explored the role of CHIT1 in cellular signaling pathways to determine whether it acts protectively or adversely in different disease states. Lee *et al.* demonstrated that CHIT1 is involved in interleukin (IL)-13 and bleomycin-induced lung fibrosis, with CHIT1^−/−^ mice showing reduced fibrosis and CHIT1-overexpressing mice exhibiting increased fibrosis, which was mediated by enhanced transforming growth factor (TGF)-β1 signaling and receptor expression.^75^ An *in vitro* study by Wang *et al.* showed that CHIT1 increases TGF-β1-induced Smad signaling and amyloid β (Aβ) phagocytosis in microglia, suggesting a protective role in Aβ clearance and Alzheimer's disease.^76^ Hong *et al.* depicted that CHIT1 plays a protective role in allergic inflammation by inhibiting T helper 2 (Th2) responses while enhancing anti-inflammatory cytokines and Treg accumulation through the regulation of TGF-β1 signaling.^77^ On the other hand, Wiesner *et al.* reported that in response to pulmonary fungal infection, recognition and cleavage of chitin *via* CHIT1 enhanced harmful Th2 cell accumulation and inflammation.^78^

### Development of small-molecule CHIT1 inhibitors

**Fig. 8:**
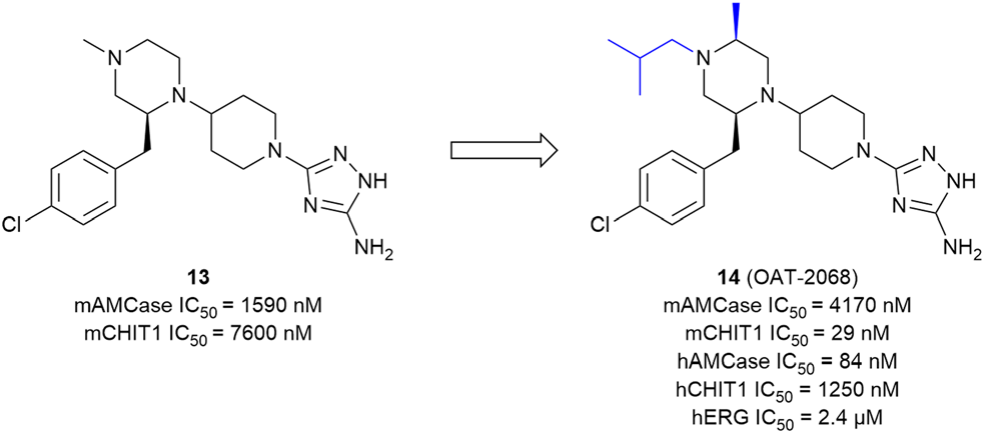
Development of the selective mCHIT1 inhibitor 14.

**Fig. 9:**
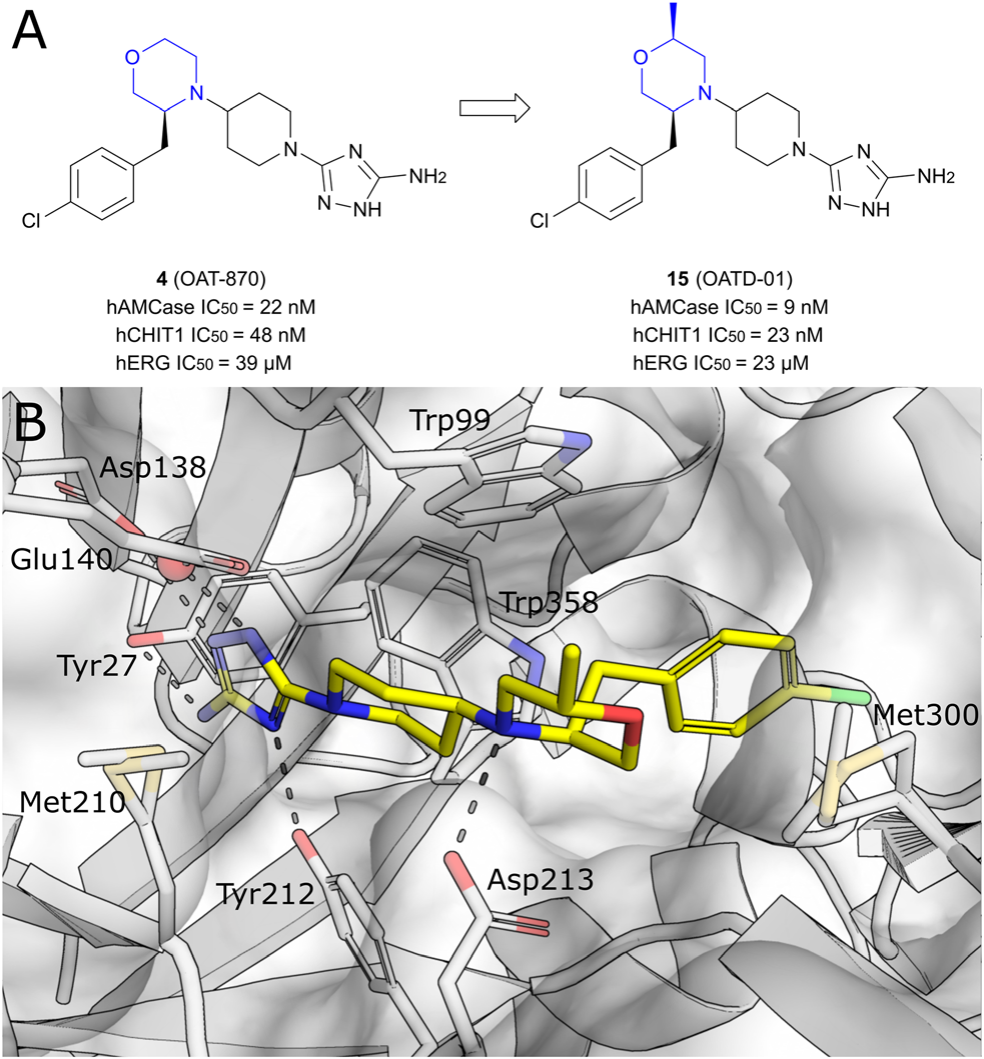
A) Optimization of non-selective chitinase inhibitor 4 towards clinical candidate 15. B) X-ray crystal structure of 15 in complex with CHIT1 (PDB ID 6ZE8).

The development of small-molecule inhibitors that selectively block CHIT1 activity could provide insights into the enzyme's role in disease progression and deliver potential treatment options. Starting with the moderately potent and unselective murine CHIT1 (mCHIT1) and AMCase (mAMCase) inhibitor 13, which was identified as part of the previously developed AMCase-targeted aminotriazole series, Mazur *et al.* aimed to develop potent mCHIT1 selective compounds.^79^ SAR studies demonstrated that the substitution pattern on the piperazine ring governs selectivity between mCHIT1 and mAMCase. Incorporating a bulky isobutyl group at the *N*1 position enhanced selectivity for mCHIT1 without compromising binding affinity. This modification resulted in the highly potent mouse CHIT1 inhibitor 14 (OAT-2068) displaying an IC_50_ value in the low nanomolar range and a 143-fold selectivity for mCHIT1 over mAMCase (Fig. 8). Molecular docking experiments revealed that the isobutyl chain extends into an aliphatic sub-pocket of mCHIT1, where it establishes enhanced hydrophobic interactions. In contrast, this hydrophobic group is less effectively accommodated within the putatively more polar region of the pocket in mAMCase. 14 demonstrated promising PK properties in single dose PK studies with an oral bioavailability of 61%. However, 14 showed inverse selectivity for the human chitinase analogs, with a 19-fold preference for hAMCase over hCHIT1.^48^ The compound was ultimately not pursued further due to high off-target activity.^80^ Koralewski *et al.* took inspiration from the previously described dual chitinase inhibitor 4 (OAT-870, see above)^47^ intending to improve off-target activity and PK properties.^80^ The primary concern with 4 was its high activity against dopamine transporters (IC_50_ = 370 nM). SAR studies revealed that introducing alkyl substituents to the morpholine ring could immediately abrogate this off-target activity while simultaneously enhancing potency against chitinases. The introduction of a “magic methyl” group in this position (→15, OATD-01) gave the most favorable on-/off-target activity profile. The PK profile determined for 15 was favorable, including high oral bioavailability in multiple species (77% to 107%) and low renal clearance. From the crystal structure of the CHIT1 complex with 15, it becomes apparent that the binding mode closely resembles the binding mode of 3 (Fig. 5B). In this case, the 2-methylmorpholine ring establishes additional van der Waals interactions with Arg269, arresting it in a single conformation, and also interacts with the side chain of Met300 (Fig. 9B). These additional interactions are likely responsible for the observed increase in potency (hCHIT1 IC_50_ = 23 nM, hAMCase IC_50_ = 9 nM) compared with the parent molecule 4. The authors demonstrated that 15 substantially alleviated the degree of lung fibrosis and reduced plasma chitinolytic activity in a murine bleomycin-induced pulmonary fibrosis model. Following these findings, 15 has already progressed through phase I trials and is currently being evaluated for pulmonary sarcoidosis in a phase II clinical trial.^6,80^ In a recent study, 15 was also evaluated as a therapeutic for chronic asthma, demonstrating significant antifibrotic effects through dose-dependent inhibition of TGF-β1 release in a mouse model of house dust mite (HDM)-induced allergic airway inflammation.^81^ Notably, the authors concluded that CHIT1 plays a significant role in airway remodeling and fibrosis, as macrophage-specific CHIT1 localization in the lungs becomes predominant in the later stages of HDM administration, highlighting its potential as a therapeutic target in severe asthma. However, given the involvement of AMCase in early allergic inflammation^45^ and the lack of selectivity of 15, it remains unclear whether the observed effects are solely attributable to CHIT1 inhibition or if AMCase inhibition also contributes, warranting further investigation to distinguish these effects.

**Fig. 10:**
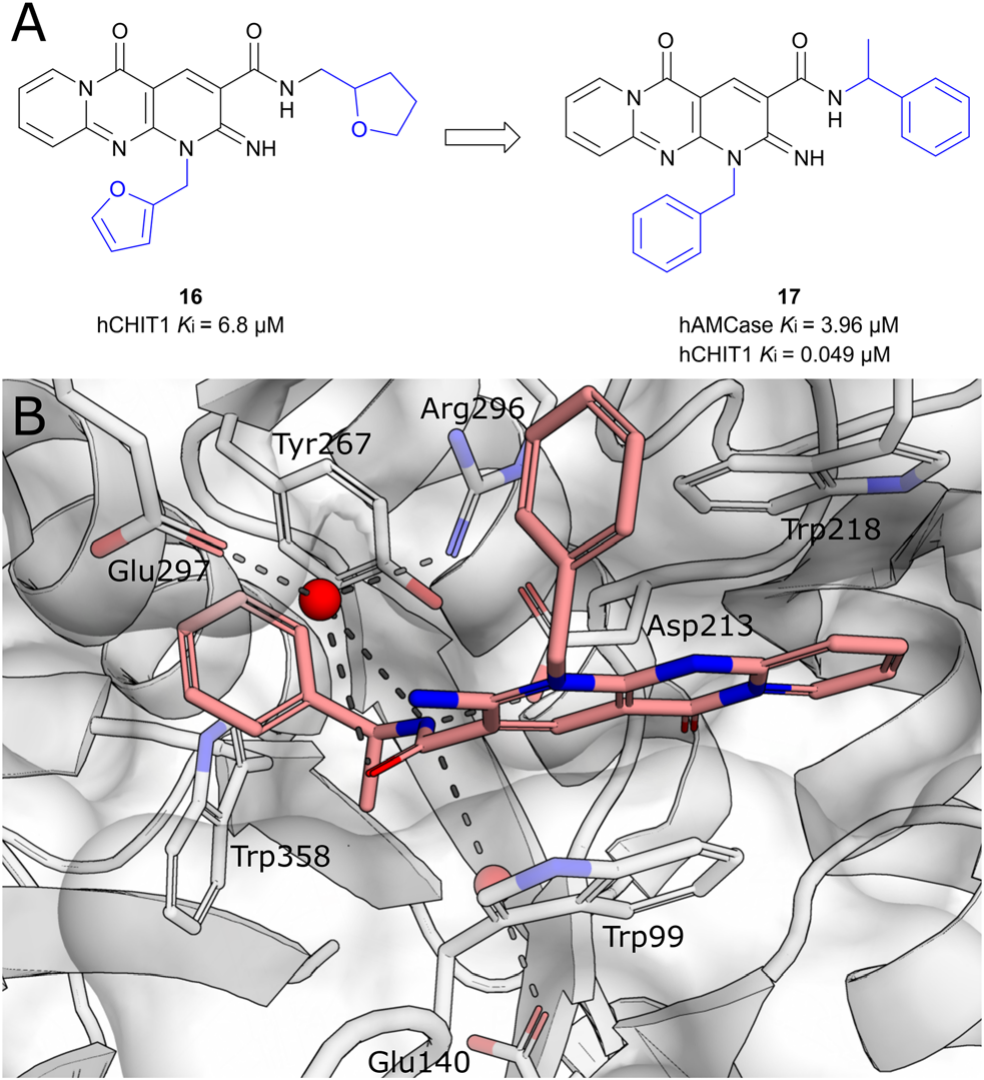
A) Structure of dipyrido-pyrimidine analog 16 and optimization towards selective high-affinity CHIT1 inhibitor 17. B) X-ray crystal structure of 17 in complex with CHIT1 (PDB ID 6JJR).

In a recent study by Jiang *et al.*, a new series of chitinase inhibitors featuring a shared dipyrido-pyrimidine-based scaffold was identified using a hierarchical virtual screening approach (Fig. 10A).^82^ Among the structural analogs of the initial hit compound 16, 17 demonstrated the strongest inhibitory effect on hCHIT1, with a *K*_i_ value in the nanomolar range and approximately 80-fold selectivity against hAMCase. Crystal structures of the complexes revealed that the key determinant for binding is the engagement of sandwiched π–π stacking interactions between the dipyrido-pyrimidine moiety with aromatic residues (Trp99 and Trp218) lining the +1 and +2 subsites (Fig. 10B). Interestingly, 17 does not extend deeply into the −1 pocket and, consequently, does not interact with the catalytic acidic residues. An additional hydrogen bond is formed by Asp213 with the 3-carbamoyl nitrogen atom. Further decoration of the dipyrido-pyrimidine scaffold with aromatic groups enabled the formation of hydrophobic π–π stacking interactions with aromatic residues (Tyr267 and Trp358) around the pocket. This led to enhanced potency (*K*_i_ = 49 nM) relative to the parent molecule 16. The observed selectivity of 17 for hCHIT1 is attributed to the substitution of His269 in hAMCase for Arg269 in hCHIT1. The guanidine group of Arg269 forms a water-mediated hydrogen bond with 17, an interaction absent in hAMCase. Consequently, the positioning of the dipyrido-pyrimidine ring and other aromatic moieties for π–π stacking with tryptophan residues lining the binding site is less optimal in hAMCase. In addition, the lower binding affinity of dipyrido-pyrimidine ligands for hAMCase compared to hCHIT1 may also stem from the smaller binding pocket of hAMCase, which reduces protein–ligand contacts and restricts the rotation of functional groups necessary for optimal interactions. The inhibitory activity of compound 17 was also tested against murine chitinases and potential off-targets, revealing similar potency but only 15-fold selectivity for mCHIT1, along with near-complete inhibition of hERG and the phosphodiesterase enzyme PDE4D2 at 10 μM, raising potential safety concerns for therapeutic use. Furthermore, the *in vivo* efficacy of compound 17 was evaluated in a bleomycin-induced lung fibrosis murine model. While the treatment of bleomycin combined with 17 effectively reduced lung fibrosis, it also triggered increased lung inflammation, indicating that chitinases could have a protective function.^76^

## Chitinase-3-like protein 1

**Fig. 11:**
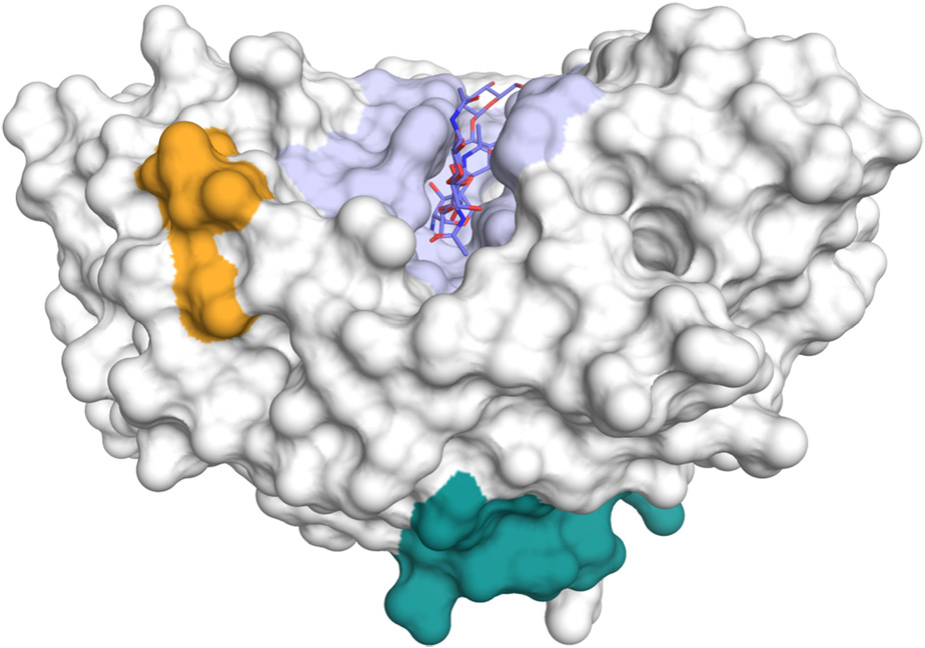
Potential heparin binding sites of CHI3L1 (PDB ID 1NWT). The chitin binding site is colored in light blue. Potential heparin binding sites spanning residues 144–147 (orange) or 334–345 (green) are located in distal positions.

The CLP chitinase-3-like protein 1 (CHI3L1), also known as YKL-40 – a reference to the first three *N*-terminal amino acids in its sequence and its molecular mass – or human cartilage glycoprotein 39 (HCgp-39), was first identified in the culture medium of MG-63 human osteosarcoma cells, alongside its close analog chitinase-3-like protein 2 (CHI3L2) or YKL-39.^83^ In addition, CHI3L1 is produced by many immune cell types, most prominently macrophages, endothelial cells, as well as many tumor cells.^17^ Despite its high similarity with AMCase and CHIT1, two mutations in the catalytic motif (Fig. 1B) prevent hydrolysis of chitin and render CHI3L1 catalytically inactive. Despite the loss of catalytic activity, CHI3L1 still retains its affinity towards chitin. Contrary to this notion, however, a recent study suggested that the catalytic inactivity of CHI3L1 is not only a result of the mutated catalytic motif in the −1 subsite but also stems from variations in non-catalytic residues.^84^ Accordingly, a mere reversion of the catalytic consensus sequence was not sufficient to restore catalytic activity. Introduction of two additional key mutations based on the CHIT1 sequence, I61M and W69T, however, resulted in chitinase activity, whereas the reverse process, introducing M61I and T69W mutations into the CHIT1 sequence, abolished its catalytic activity. In addition to chitin, major components of the extracellular matrix, such as heparin/heparan sulfate,^85^ hyaluronic acid,^17^ and collagen,^86^ have been identified as natural ligands of CHI3L1. However, the respective binding mechanisms and functional consequences of these interactions are still elusive and a matter of ongoing discussion. The putative glycosaminoglycan (GAG) binding site of CHI3L1 is located on the extended surface of the protein. Initially, a consensus heparin-binding motif (RRDK) in positions 144–147, located in relative proximity to the chitin-binding grove, was assumed to convey heparin binding affinity (Fig. 11).^85,87^ It was later shown, however, that a different KR-rich domain on the distal end of the protein in positions 334–345 enabled heparin binding.^85^ As a key regulator of innate immunity, CHI3L1 plays a crucial role in the defense against pathogens,^88,89^ inflammation,^90–93^ as well as for tissue repair and remodeling.^94,95^ In addition, overexpression of CHI3L1 is considered as a marker of tumor cells.^92,96–99^

In healthy individuals, serum levels of CHI3L1 can range from 0 to 50 ng mL^−1^. During inflammation, cardiovascular diseases, fibrosis, cancer, neurodegenerative diseases, or parasitic invasions, the serum level of CHI3L1 can increase to more than 100 ng mL^−1^.^100^ Elevated levels of CHI3L1 are often correlated with disease progression, particularly in cancer. This correlation was also shown in amyotrophic lateral sclerosis (ALS) patients, for which CHI3L1 levels can be used to predict disease severity and survival rate.^101,102^ Additionally, it was found that alcohol consumption can affect CHI3L1 levels. Here, CHI3L1 expression is associated with an increased risk of ischemic stroke-specific mortality in drinkers.^103^ Overall, the involvement of CHI3L1 in many different diseases gave rise to countless studies investigating the protein as a potential biomarker or therapeutic target.^96,98,103–105^

Another example for the complex relationship between CHI3L1 expression and the immune system stems from studies on gallbladder cancer.^92,106^ Elevated CHI3L1 levels in plasma and tissue were found to correlate with tumor size and metastasis formation. CHI3L1, mainly produced by infiltrating M2 macrophages in the tumor microenvironment, induced expression and secretion of growth differentiation factor 15 (GDF15) in tumor cells. This ultimately causes an imbalance of pro and anti-PD-L1 regulation, which enhances PD-L1 expression, inhibits T-cell cytotoxicity, and results in tumor immune evasion.

The biological functions of CHI3L1 at the cellular level are fully independent of chitin recognition and can be categorized as 1) stimulation of cell growth, proliferation, and survival through the activation of major signal transduction pathways, *e.g.*, MAPK/Erk and PI3K/Akt. 2) Activation and differentiation of immune cells, influencing, for example, macrophage maturation and T helper cell polarization. 3) Regulation of ECM synthesis/degradation, which, in turn, affects tissue remodeling in injury and fibrosis, as well as invasive cancer progression. These effects are mediated by various cellular receptors of CHI3L1, with Interleukin-13 receptor subunit alpha-2 (IL-13Rα2) assumed to be its main receptor.^107,108^ The formation of a complex between IL-13Rα2, CHI3L1, and either IL13 or TMEM219 results in the activation of the MAPK/Erk, Akt, and Wnt/β-catenin signaling pathways.^17,109^ For an in-depth discussion of the pleiotropic biological functions of CHI3L1 and its presumed receptors, we refer to recent reviews on the topic elsewhere.^17,110^

**Fig. 12:**
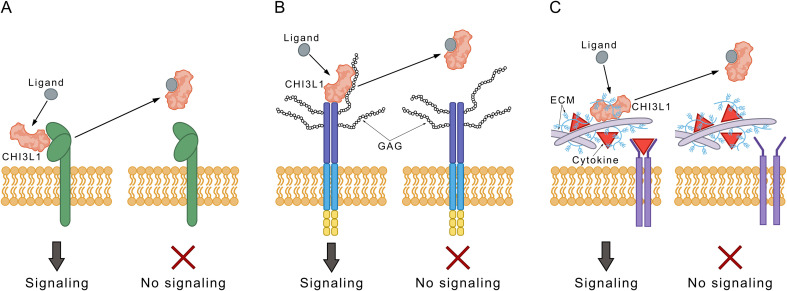
Mechanistic hypotheses of the effect of small-molecule ligands of CHI3L1 on its cellular effector functions. A) Direct competition with CHI3L1–receptor interactions. B) Competition with glycosaminoglycan (GAG)-mediated interactions. C) Modulation of cytokine reservoirs in the extracellular matrix by liberation of bound CHI3L1. Based on ref. 109. Copyright 2024 American Chemical Society.

The exact nature of the interaction between CHI3L1 and its receptors on the molecular level has not been elucidated yet. Currently, three main hypotheses are under consideration: 1) a direct protein–protein interaction between CHI3L1 and its receptors; 2) a GAG-mediated interaction between CHI3L1 and its receptors; 3) the liberation of cytokine reservoirs in the extracellular matrix after displacement by CHI3L1 without a direct receptor interaction. Importantly, it is generally assumed that the signaling functions of CHI3L1 are sensitive to small molecule ligands binding to the chitin-binding site (Fig. 12).

The exact mechanism, however, in which small molecule CHI3L1 ligands modulate its cellular effector functions, is yet unclear.

### Development of therapeutic antibodies targeting CHI3L1

Antibody-based approaches have been investigated for modulating dysregulated CHI3L1 functions. The murine anti-CHI3L1 antibody mAY was shown to inhibit angiogenesis in an *in vitro* tumor model by interrupting angiogenic signaling cascades through the vascular endothelial growth factor receptor 2 (VEGFR 2) and the MAPK/Erk pathway.^111^ In addition, a reduction of radiotherapy resistance mechanisms mediated by PI3K/Akt was observed after mAY application. An investigation of mAY in a mouse xenograft model of glioblastoma showed that neutralization of CHI3L1 significantly inhibited tumor growth and metastasis formation in some animals, however, mAY failed to provide full protection in all treated mice. By replacing the complementarity-determining regions (CDRs) of human IgG1 with the mAY-CDR by *in silico* CDR-grafting, the humanized anti-CHI3L1 antibody rosazumab was developed.^94^ Rosazumab was found to target the KR-rich region at residues 334–347, a putative heparin binding site, with high affinity (*K*_D_ = 46 nM). The antibody elicited a direct cytotoxic effect by modulating the anti-apoptotic β-catenin pathway.^112^ To investigate its effect on cell migration and tumor vascularization, rosazumab was tested against glioblastoma and gallbladder cancer cell lines *in vitro*. Here, the antibody displayed a significant inhibitory effect on cell migration and tube formation of CHI3L1 expressing tumor cells. *In vivo* studies in a murine xenograft model provided further support for the notion that rosazumab significantly reduces tumor growth and angiogenesis while promoting tumor cell apoptosis. These studies highlight the value of anti-CHI3L1 antibodies, such as rosazumab and mAY, as potential therapeutic agents to combat tumor angiogenesis, metastasis, and immunosuppression.

A bi-specific antibody against CHI3L1 and PD-1, dubbed FRGxPD-1, displaying high affinity to both proteins (*K*_D_ = 1 nM) was tested in an *in vitro* melanoma model.^92^ Here, a synergistic cytotoxic effect of FRGxPD-1 compared with either anti-CHI3L1, anti-PD-1, or a combination of both individual antibodies was observed. The effect was mediated by an increased activation of CD8+ cytotoxic T cells, as well as an enhancement of the expression of the tumor suppressor PTEN in treated cells. In an *in vivo* model of pulmonary melanoma metastasis, FRGxPD-1 was similarly found to synergistically reduce the number of pleural colonies in treated animals.

### Chitin oligosaccharides as potential CHI3L1 modulators

The hypothesis that the immunomodulatory properties of chitin or COS, which are mediated by chitin sensing proteins, can be exploited for therapeutic purposes has a long tradition. In 1986, Suzuki *et al.* investigated the effect of hexa-*N*-acetylchitohexaose and chitohexaose in murine tumor models Both COS elicited a tumor-suppressive response that was attributed to an activation of the host immune system rather than a direct cytotoxic effect.^113^ Yet, no information about the mechanism of action was available at that time.

This notion prompted Libreros *et al.* to hypothesize that the beneficial effects of chitin treatment in tumor models could originate from its interaction with CHI3L1.^114^ It was found that the intraperitoneal application of chitin microparticles to mammary tumor-bearing mice resulted in a downregulation of the pro-inflammatory mediators C-chemokine ligand 2 (CCL2), CXC motif chemokine ligand 2 (CXCL2) and matrix metalloproteinase 9 (MMP-9), whereas the production of interferon γ was markedly increased. In addition, a significant downregulation of CHI3L1 itself upon chitin treatment was observed, suggesting a negative feedback loop in the regulation of chitin sensing pathways. Treated mice displayed a significant reduction of tumor volume and lung metastasis formation. Even though CHI3L1 was found to co-localize with chitin particles, the observed effect could not be unambiguously attributed to a modulation of CHI3L1 signaling.

In a model of Lewis lung carcinoma, COS treatment of tumor-bearing mice similarly resulted in a reduction of CHI3L1 plasma levels, although no effect on tumor growth was observed.^115^ In combination with immune checkpoint inhibition, however, COS treatment resulted in a synergistic delay of tumor growth. Similar effects of chitin treatment on tumor immunosuppression and lymphatic metastasis were observed in a murine triple-negative breast cancer model.^116^ Here, chitin treatment resulted in a significant reduction of primary tumor progression through downregulation of CLPs originating from tumor-associated neutrophils. Reduced numbers of immunosuppressive cell types and increased numbers of anti-tumorigenic T cells were observed in the primary tumor as well as axial lymph nodes. In addition, chitin treatment alleviated anti-PD-1 resistance in combination with immune checkpoint blockade.

It has to be considered that chitin is a heterogeneous polymer that can differ in degree of polymerization, degree of acetylation, and pattern of acetylation. Very little information about the impact of the fine structure of chitin on the binding affinity to CHI3L1 is currently available. The only account in literature is a study of Einarsson *et al.*, who determined lower affinities for partially acetylated COS, compared with the fully acetylated chitin hexamer.^117^

Due to the pleiotropic effects of chitin and COS on immune functions, definite evidence of CHI3L1 modulation by small molecule ligands is still lacking, highlighting a unique opportunity for medicinal chemistry to provide potent and selective chemical probes targeting CHI3L1. This research, however, is still in its infancy.

### Development of small-molecule CHI3L1 modulators

**Fig. 13:**
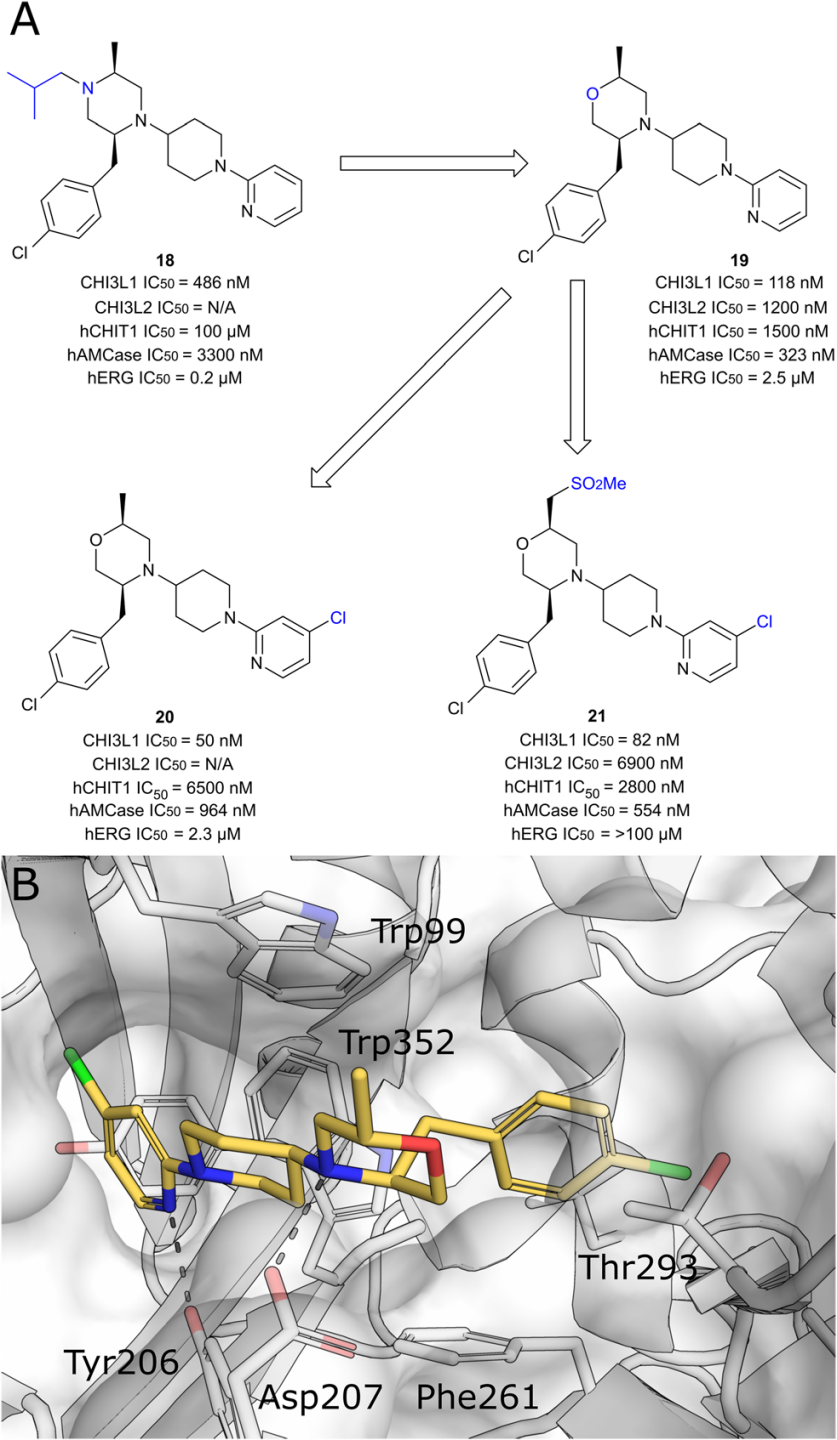
A) Discovery of morpholine-based CHI3L1 ligands. B) Crystal structure of CHI3L1 in complex with 20 (PDB ID 8R4X).

In an extension of their portfolio of AMCase and CHIT1 inhibitors, the Polish biotech company Molecure recently published an extensive study, in which the development of selective CHI3L1 ligands was disclosed (Fig. 13A).^118^ After screening an internal library of 500 chitinase inhibitors using a microscale thermophoresis assay, five compounds with *K*_D_ values in the μM range were identified as potential starting points. Interestingly, the aminotriazole pharmacophore of previously identified AMCase/CHIT1 inhibitors turned out to be detrimental, because key interactions with the active site residues of functional chitinases could not be formed in the inactive CLP (see Table 2 for a summary of all discussed CHI3L1 modulators). Instead, the screen identified compounds featuring less polar and weakly basic heterocycles such as pyridine (→18), thiazole or isoxazole in this position. In the respective crystal structures, these substitutions effectively filled the hydrophobic −1 pocket of CHI3L1, while simultaneously accepting a hydrogen bond from Tyr206. Additional hydrophobic interactions in the chitin binding site, as well as CH–π interactions with Trp99 and Trp352, further contributed to binding affinity. Further hit optimization efforts yielded morpholine derivative 19, which displayed higher CHI3L1 affinity and reduced inhibition of CHIT1 and hERG. The introduction of a chloro-substituent on the pyridine moiety further improved affinity and selectivity, however, the resulting compound 20 showed a concerning propensity for hERG-mediated off-target toxicity. To tackle this unfavorable property, a polar methyl sulfone function was introduced (21), which fully abrogated hERG activity. From the crystal structure of the CHI3L1 complex with 20 (Fig. 13B) and supporting simulations, the authors concluded that hydrogen bonds with Tyr206 and Asp207, as well as hydrophobic interactions withTrp352, Thr293, and Phe261 were key interactions to restrict the movement of the flexible Trp99 sidechain and achieve high affinity to the protein. As a result, the application of previous experience with chitinase inhibitors successfully yielded potent and selective ligands for CHI3L1, whose main drawback was the moderate PK profile with bioavailabilities of 6%, 18%, and 31% for 18, 20, and 21, respectively.

**Table 2:** Summary of biological assay data for CHI3L1 ligands

Compound	CHI3L1 IC_50_ [nM]	CHI3L2 IC_50_ [nM]	hAMCase IC_50_ [nM]	hERG IC_50_ [μM]	Ref.
18	486	N/A	3300	0.2	118
19	118	1200	323	2.5	118
20	50	N/A	964	2.3	118
21	82	6900	554	≥100	118

A critical observation about the ligand binding properties of CHI3L1 was made in a competitive assay format recording competition between the heparin and chitin binding sites. A probe selective for the chitin binding site could be fully displaced by a chitin oligosaccharide, whereas the addition of heparin-derived polymers only resulted in the partial displacement of the probe. Similarly. a heparin-based probe was only partially displaced by compounds binding to the chitin binding site. The authors concluded that the respective binding sites must partially overlap. Based on the previous elucidation of distal heparin binding sites, however, an allosteric communication between the two binding sites seems also possible.

**Fig. 14:**
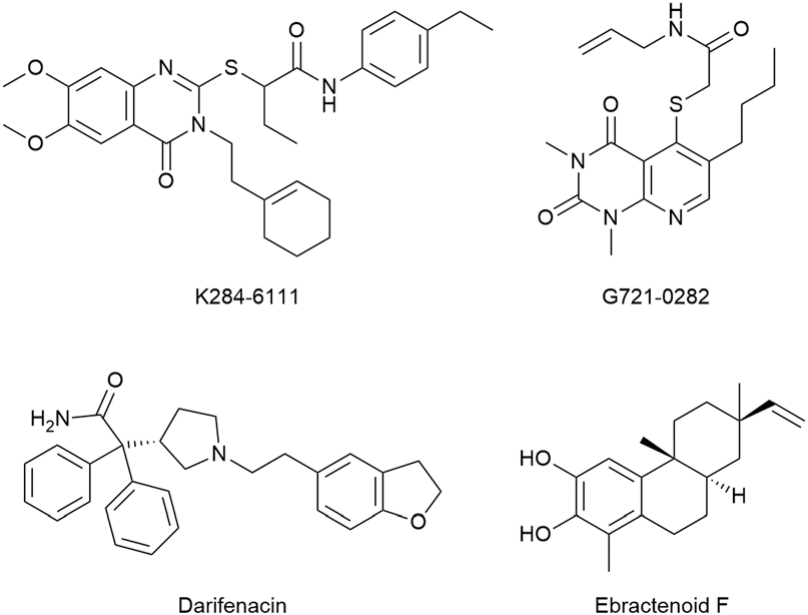
Chemical structure of putative CHI3L1 ligands from virtual screening campaigns.

Other reports claimed the discovery of potential CHI3L1 ligands originating from virtual screening hits. Three of those hits, K284-611, G721-0282, and ebractenoid F (Fig. 14) were then directly progressed into fairly complex cellular and murine disease models of lung metastasis formation,^119^ atopic dermatitis,^93^ lung cancer,^120^ or osteosarcoma,^121^ albeit without unequivocal evidence of target engagement or an experimental validation of the binding mode inferred from docking studies. To repurpose known drugs and drug candidates, a library of such compounds was screened for CHI3L1 binding by molecular docking.^122^ This effort identified the muscarinic receptor antagonist darifenacin as a potential CHI3L1 ligand, even though neither experimental evidence of the interaction, nor a validation of the predicted binding mode was presented. This compound was then evaluated for activity in pancreatic ductal adenocarcinoma models.

## Conclusion

Due to their pleiotropic cellular effector functions at the intersection of important regulatory pathways in host defense, immune homeostasis, cellular proliferation, and tissue remodeling, human chitinases and CLPs are prime targets for pharmacotherapy. Due to the high structural similarity of human GH18 chitinases, the most advanced candidates for the targets discussed here rely on a mostly conserved pharmacophore targeting the −1 subsite. As a result, selectivity against related GH18 proteins remains a challenge in some cases. This highlights the potential of drug-like small molecules, which can be more easily optimized in terms of potency, selectivity, and pharmacokinetics, in comparison to COS or peptide-derived natural chitinase inhibitors. Despite tremendous progress in this area, it has not yet been elucidated how small molecules binding to the chitin binding site can modulate the non-chitin-related functions of GH18 proteins, highlighting the need for further studies. Nevertheless, the first compounds targeting human chitinases and CLPs have advanced towards (pre-)clinical development, paving the way for a new class of therapeutics to alleviate inflammatory conditions, fibrosis, and cancer.

## Data availability

No primary research results, software, or code have been included and no new data were generated or analysed as part of this review.

## Author contributions

Önder Kurç and Nick Rähse – writing (original draft), visualization. Jonathan Cramer and Holger Gohlke – writing (review and editing), funding acquisition, supervision.

## Conflicts of interest

There are no conflicts to declare.
